# Characterization and Pathogenicity of *Fusarium* Species Associated with Soybean Pods in Maize/Soybean Strip Intercropping

**DOI:** 10.3390/pathogens8040245

**Published:** 2019-11-19

**Authors:** Muhammd Naeem, Hongju Li, Li Yan, Muhammad Ali Raza, Guoshu Gong, Huabao Chen, Chunping Yang, Min Zhang, Jing Shang, Taiguo Liu, Wanquan Chen, Muhammad Fahim Abbas, Gulshan Irshad, Muhammad Ibrahim Khaskheli, Wenyu Yang, Xiaoli Chang

**Affiliations:** 1 College of Agronomy, Sichuan Agricultural University, Chengdu 611130, China; muhammdnaeem201@gmail.com (M.N.); Lihongjucomeon@163.com (H.L.); yanli1178@outlook.com (L.Y.); razaali0784@yahoo.com (M.A.R.); guoshugong@126.com (G.G.); Chenhuabao12@163.com (H.C.); chunping79@163.com (C.Y.); yalanmin@126.com (M.Z.); shangjing_edu@163.com (J.S.); mssiyangwy@sicau.edu.cn (W.Y.); 2 State Key Laboratory for Biology of Plant Diseases and Insect Pests, Institute of Plant Protection, Chinese Academy of Agricultural Sciences, Beijing 100193, China; liutaiguo@caas.cn (T.L.); wqchen@ippcaas.cn (W.C.); 3 Department of Plant Pathology, PMAS Arid Agriculture University, Rawalpindi 46000, Pakistan; dr.abbasmf@gmail.com (M.F.A.); gulshanirshadpp@gmail.com (G.I.); 4 Department of Plant Protection, Faculty of Crop Protection, Sindh Agriculture University, Tandojam 70060, Pakistan; mikhaskheli@sau.edu.pk

**Keywords:** soybean (*Glycine max* L.), maize/soybean strip intercropping, pod decay, *Fusarium* species, diversity

## Abstract

Intercropping has been considered as a kind of a sustainable agricultural cropping system. In southwest China, maize/soybean strip intercropping has commonly been practised under local limited agricultural land resources. However, heavy rainfall in combination with high humidity and low temperatures cause severe pod and seed deterioration in the maturity and pre-harvesting stages of intercropped soybean. Numerous *Fusarium* species have been reported as the dominant pathogens of soybean root rot, seedling blight, as well as pod field mold in this area. However, the diversity and pathogenicity of *Fusarium* species on soybean pods remain unclear. In the current study, diseased soybean pods were collected during the cropping season of 2018 from five different intercropped soybean producing areas. A total of 83 *Fusarium* isolates were isolated and identified as *F. fujikuroi*, *F. graminearum*, *F. proliferatum*, and *F. incarnatum*-equiseti species complex based on morphological characteristics and phylogenetic analysis of the nucleotide sequence of *EF1-α* and *RPB2* genes. Pathogenicity tests demonstrated that all *Fusarium* species were pathogenic to seeds of the intercropped soybean cultivar Nandou12. *Fusarium fujikuroi* had the maximum disease severity, with a significant reduction of seed germination rate, root length, and seed weight, followed by *F. equiseti*, *F. graminearum*, *F. proliferatum*, and *F. incarnatum*. Additionally, the diversity of *Fusarium* species on soybean pods was also considerably distinct according to the geographical origin and soybean varieties. Thus, the findings of the current study will be helpful for the management and resistance breeding of soybean pod decay in the maize/soybean intercropping system.

## 1. Introduction

Soybean (*Glycine max* L.) is an economically-important oilseed crop and is considered a critical processing material for supplementary nutritious foods [1,2]. China has a long history of soybean domestication, where the plant has been cultivated since ancient times [3,4]. Currently, China is the world’s largest soybean consumer, with imports recorded at 82.31 million tons in 2018 (Ministry of Agriculture of People’s Republic of China, http://www.moa.gov.cn/). According to a Chinese government report, the cultivated area of soybean is expected to increase by about 10 million hectares by 2020 due to the rising pressure of domestic demand for soybean products. In southwest China, soybean is presently cultivated in the maize/soybean strip intercropping system as limited solar, thermal, and land resources are available [5,6]. This intercropping pattern is usually comprised of two-row maize spaced with two-four rows of soybean as each strip unit. Previous studies have demonstrated that in contrast to monoculture, maize/soybean strip intercropping has distinct advantages in terms of promoting resource use efficiency [7,8,9,10,11], increasing crop production [12,13,14,15], improving soil quality [16], suppressing field weeds [6] and maintaining the agro-ecological system [5]. Therefore, it has also been practiced in several single-season cropping regions of Huang-Huaihai and northwest China as a kind of creative, sustainable agriculture cropping pattern [5]. However, in southwest China, there is continuous rainfall (approximately 25% of the annual precipitation), low temperatures (13–21 °C), and high humidity (85–100%) in late October. This is favorable for pathogen infection and associated pod field mold and decay [17,18,19]. Infected pods often lead to seed decay and seed rot, resulting in drastic yield losses and seed contamination [20]. This may ultimately reduce crop productivity significantly, and also represent a potential risk for the application of maize/soybean intercropping. 

Previous studies have demonstrated that diverse species of *Fusarium* can infect soybean at almost every growth stage, causing seedling blight [21,22,23], pod and seed decay [20], and root rot [24,25]. Furthermore, it is also evident that a complex of *Fusarium* species, mainly dominated by *F. oxysporum* and *F. solani* (newly named as *Neocosmospora solani*)*,* is responsible for *Fusarium* root rot in soybean across the world [24,25,26,27]. Researches have shown that the pod and seed decay caused by *Fusarium* species have resulted in reduced seed quality, less emergence, and improper seedlings growth of soybean [28,29]. Moreover, many pathogens involved in seed decay often infect soybean pods [22], for instance, with the high isolation frequencies and aggressiveness of *F. graminearum* observed on soybean pods rather than seeds [21,22,28]. Additionally, the mycotoxins and secondary metabolites produced by *Fusarium* species are also capable of contaminating pods and seeds and endangering the health of humans and animals [30,31,32,33]. Since most soybean cultivars are still lacking resistance or tolerance to *Fusarium* species [27,34,35], the control of *Fusarium*-related diseases remains problematic. At present, a combination of disease management strategies that include chemical fungicides [36,37], biological control agents, crop rotation/intercropping [38]), and reduced- or no-tillage [39] have been used on a large scale for the management of *Fusarium*-related soybean diseases. 

Although the significance of *Fusarium* species to soybean production has been widely documented in previous studies, their characterization and pathogenicity associated with soybean pods, especially with regards to intercropped soybean, have been poorly investigated. The main objectives of the current study were to characterize the diversity and geographical distribution and to compare the pathogenicity of *Fusarium* species associated with intercropped soybean pod decay in the southwest of China. Accordingly, this study will provide some supportive knowledge for a more systematic understanding of the infection relationship of *Fusarium* species with different soybean tissues and *Fusarium*-related disease management in intercropped soybean in southwest China.

## 2. Results

### 2.1. Identification of Fusarium Species Associated with Intercropped Soybean Pods

In the current study, a total of 83 *Fusarium* isolates from 102 diseased soybean pods were isolated from 16 different varieties (Table 1). *Fusarium* species were initially identified based on morphological characteristics. The characteristics of culture and colony are presented in the Supplementary Figure S1, and the features of macroconidia are described in Table 2. For further molecular verification, partial gene sequences of translation elongation factor 1-alpha (*EF1-α*) and RNA polymerase II second largest subunit (*RPB2*) were amplified, and analyses of sequence similarity showed that 83 *Fusarium* isolates exhibited above 93–99% similarity with *F. fujikuroi*, *F. graminearum*, *F. proliferatum*, and *F. incarnatum*-equiseti species complex on the databases of *Fusarium* MLST (http://www.cbs.knaw.nl/fusarium) and the FUSARIUM-ID (http://isolate.fusariumdb.org). A total of 47 isolates were identified of *F. fujikuroi*, 10 isolates of *F. proliferatum*, 3 isolates of *F. graminearum*, 21 isolates of *F. equiseti*, and 2 isolates of *F. incarnatum* (Table 1). 

**Figure 1:**
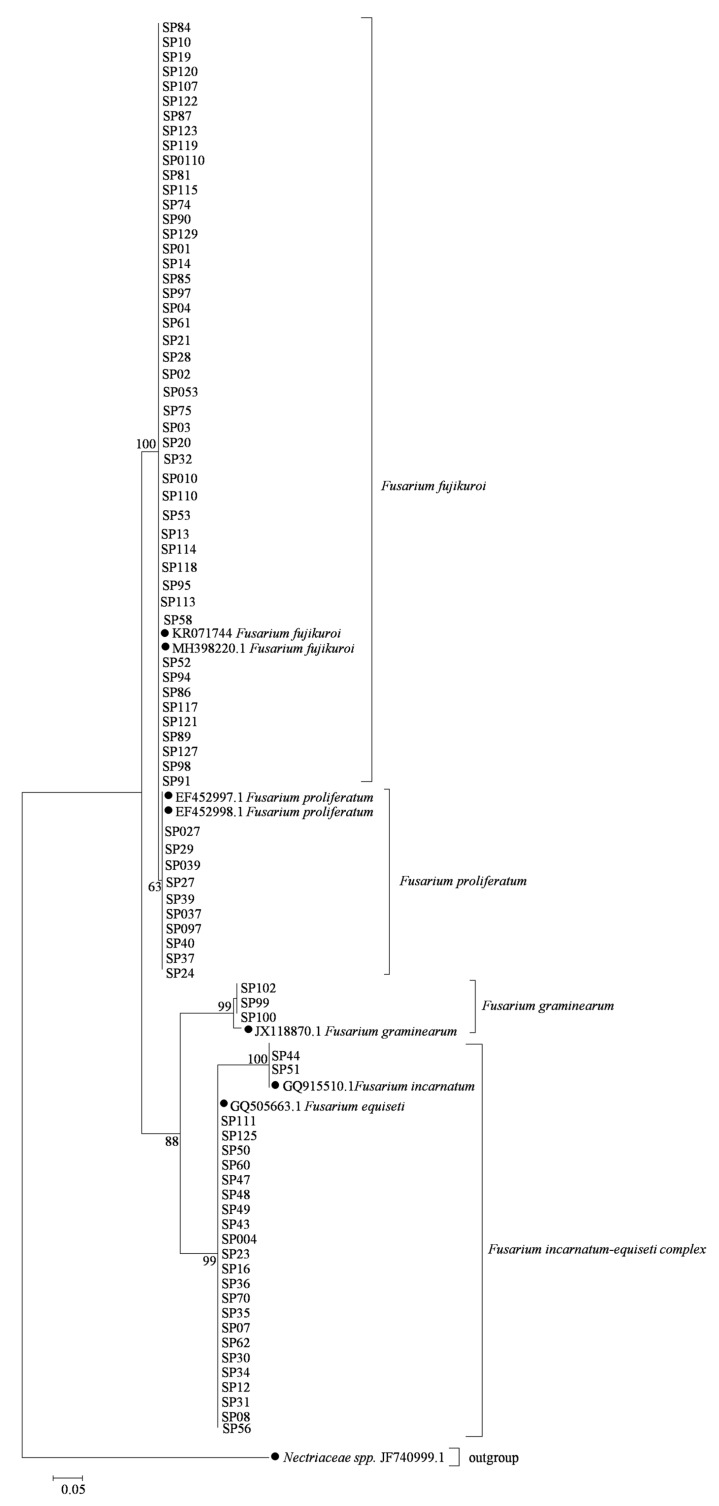
Phylogenetic tree of *Fusarium* species associated with soybean pods based on the *EF1- α* gene. A phylogenetic tree of *EF1-α* nucleotide sequences was constructed using the Maximum-likelihood method in MEGA 7.0.26 (Pennsylvania State University). Bootstrap support values were ≥50% from 1000 replications, which are shown at the nodes. *Nectriaceae spp.* (JF740999.1) was selected as an outgroup. The *EF1-α* sequences of referred *Fusarium* isolates were obtained from GenBank and are indicated by a solid dark diamond.

**Figure 2:**
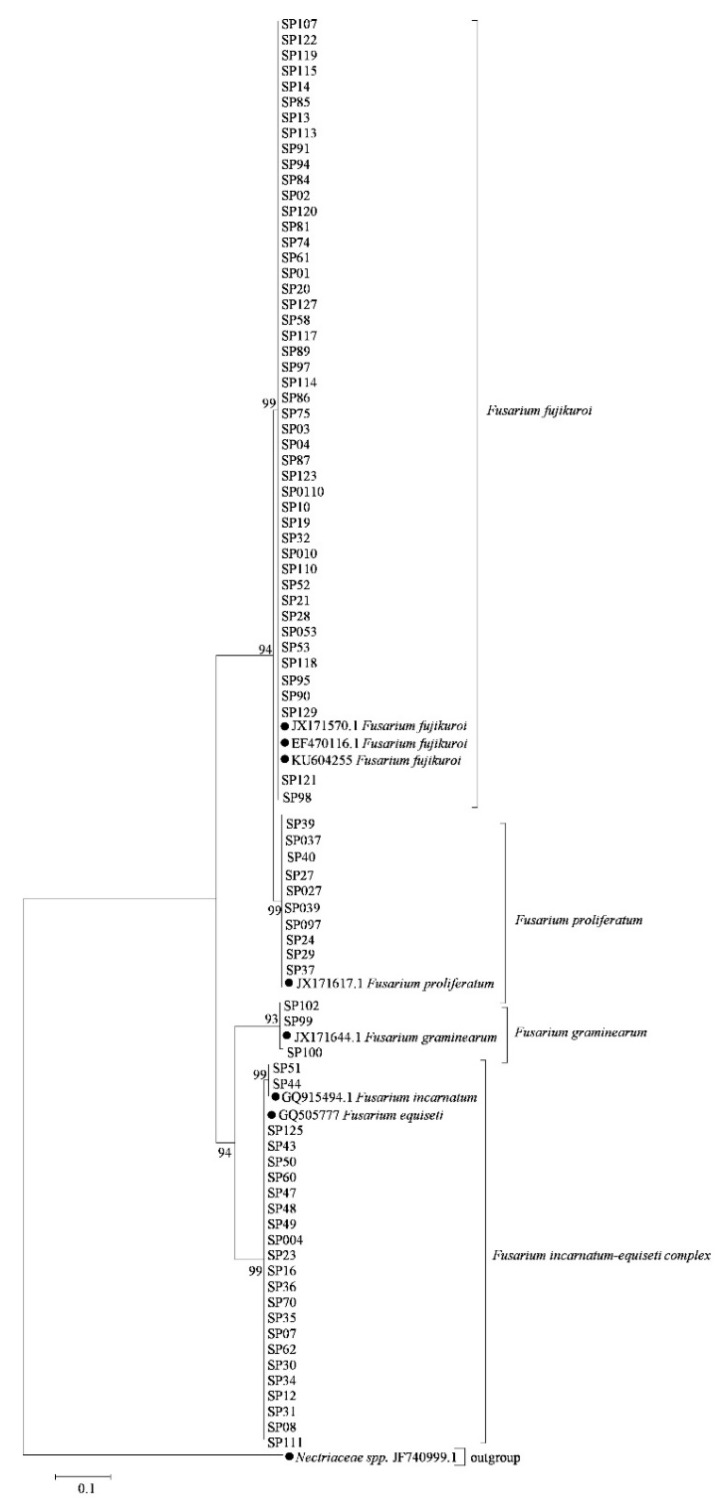
Phylogenetic tree of *Fusarium* species associated with soybean pods based on the *RPB2* gene.A phylogenetic tree of *RPB2* nucleotide sequences was constructed using the Maximum-likelihood method by MEGA 7.0.26 (Pennsylvania State University). Bootstrap support values were ≥50% from 1000 replications, which are shown at the nodes. *Nectriaceae spp.* JF740999.1 was selected as an outgroup. The *RPB2* sequences of referred *Fusarium* isolates were obtained from GenBank and are indicated by a solid dark diamond.

For the phylogenetic analysis, a maximum-likelihood tree was constructed using a total of 83 *Fusarium* isolates based on the *EF1-α* (Figure 1) and *RPB2* (Figure 2) genes, respectively, and 14 referred sequences of *Fusarium* isolates and one outgroup species *Nectriaceae spp.* from GenBank listed in Supplementary Table S1. The results showed that the phylogenetic trees constructed from *EF1-α* (Figure 1) and *RPB2* gene (Figure 2) showed a similar phylogenetic relationship of *Fusarium* species. Except for the fact that *F. incarnatum* shared a big clade with *F. equiseti*, composing one *F. incarnatum*-equiseti species complex, other species were clearly classified into a single clade (Figure 1 and Figure 2). Thus, morphological characteristics and molecular analysis revealed that all *Fusarium* isolates associated with soybean pods in Sichuan Province, China were identified as *F. graminearum*, *F. proliferatum*, *F. fujikuroi*, and *F. incarnatum*-equiseti species complex.

### 2.2. Pathogenicity Test of Fusarium Species 

**Figure 3:**
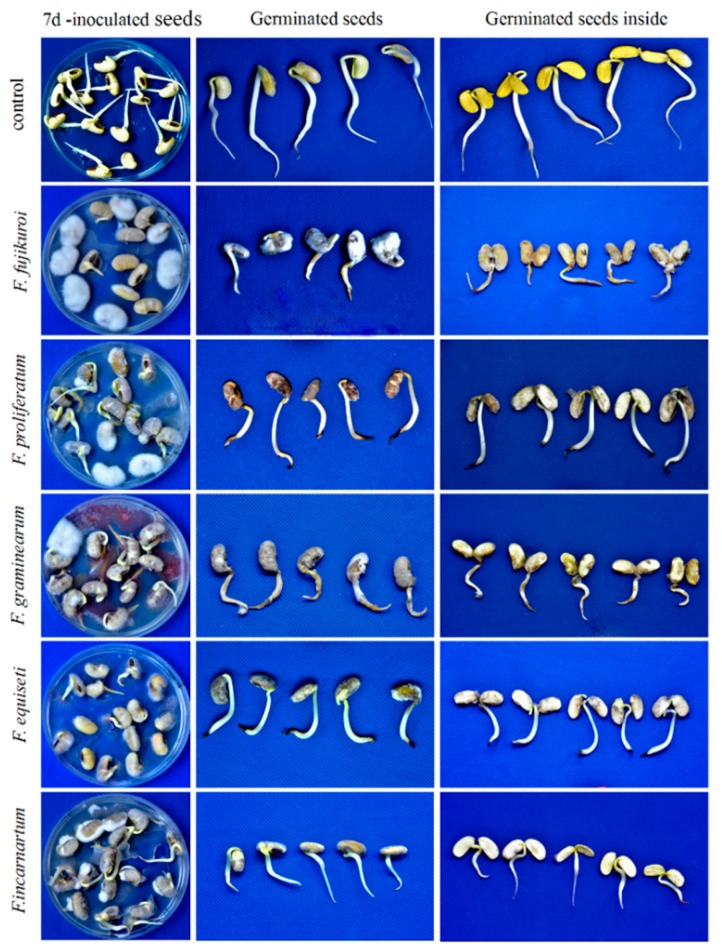
Symptoms on soybean seedlings inoculated with representative *Fusarium* isolates. Soybean seeds were inoculated with the representative *Fusarium* isolates including *F. fujikuroi* (SP39), *F. proliferatum* (SP37), *F. graminearum* (SP100), *F. equiseti* (SP30), and *F. incarnatum* (SP51) through a seed soaking inoculation method at a final concentration of 1 × 10^5^ spores per mL, and cultured on PDA medium. After 7 days of inoculation, disease symptoms were observed.

After *Fusarium* species infect a soybean pod, they often penetrate into pod coats and deteriorate the seeds, leading to a high frequency of seed decay. Therefore, in the current study, a pathogenicity test was performed on soybean seeds. After 7 days of inoculation with spore suspension, soybean seeds were covered with the mycelia of the representative *Fusarium* isolates; they showed typical rotting, softening, and browning symptoms compared to water-inoculated seeds. Our results demonstrated that different isolates of each *Fusarium* species exhibited almost no significant differences in the disease severity index (*DSI*) and the percentage of mycelium covering the area (*PMC*) (Figure 3 and Table 3). Among them, *F. fujikuroi* showed more than 70% *DSI* and 82% *PMC*, and inoculated seeds displayed the most rot, softening, and browning inside. *Fusarium equiseti* and *F. graminearum* were found to be the second most aggressive pathogens, with almost similar *DSI* and *PMC*, followed by *F. proliferatum*. *Fusarium incarnatum* in the *F. incarnatum*-equiseti species complex rarely caused soybean seeds to soften and rot, and thus, showed the lowest aggressiveness level on seeds, with a range of 24.0–26.44% for *PMC* and 35.83–40.44% for *DSI*, respectively. For negative control seeds, there were almost no disease symptoms (Figure 3).

We also assessed the relationship of the pathogenicity of five *Fusarium* species with the emergence rate, root length, and seed weight of soybean, which is presented in Table 3. Our results demonstrated that the seedling germination rate decreased when the severity of seed rot increased. Compared to the control, *F. fujikuroi* had the maximum *DSI* and *PMC* combined with a severe reduction of soybean germination rate (55–57.66%) and seed weight (0.40–0.47 g), which followed by *F. equiseti*, *F. graminearum*, and *F. proliferatum*, while *F. incarnatum* had a high germination rate, i.e., 81.66–88.33%, and the seed weight ranged from 0.73–0.76 g compared to the water-inoculated control. Fresh root length was decreased after the inoculation with *F. equiseti*, followed by *F. fujikuroi*, *F. graminearum*, *F. proliferatum*, and *F. incarnatum*. High seed weights, germination rates, and root lengths were observed in *F. incarnatum* infection when compared to other species, indicating that there is some specific relationship between these parameters and the pathogenicity of the tested *Fusarium* species.

### 2.3. Frequency of Fusarium Species Associated with Intercropped Soybean Pods

**Figure 4:**
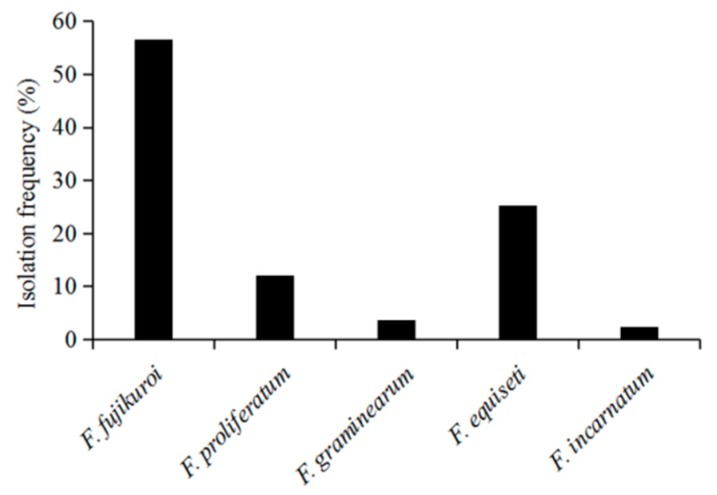
Isolation frequency of *Fusarium* species from soybean pods in the maize/soybean strip intercropping. *Fusarium* species were isolated and purified on PDA medium, and the percentage of isolates obtained for each species of *Fusarium* was calculated and used as the isolation frequency.

As shown in Figure 4, the isolation frequency of each identified *Fusarium* species associated with the intercropped soybean pods was different. Among them, *F. fujikuroi* and *F. equiseti* had the highest isolation frequencies, i.e., 56.6% and 25.3%, respectively, followed by *F. proliferatum* with 12%, whereas *F. graminearum* and *F. incarnatum* had the lowest among the identified *Fusarium* species. Thus, *F. fujikuroi* was found to be the dominant *Fusarium* species associated with intercropped soybean pod decay in Sichuan Province, China.

### 2.4. Diversity of Fusarium Species on Intercropped Soybean in Association with Geographical Origin and Soybean Varieties

**Figure 5:**
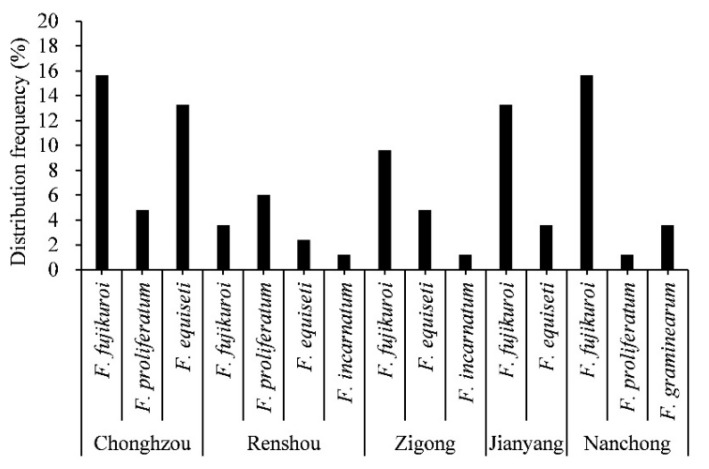
Distribution frequency of *Fusarium* species in different intercropped soybean-producing areas of Sichuan Province, China. The percentage of each species of *Fusarium* from five intercropped soybean-growing areas, i.e., Chongzhou, Renshou, Nanchong, Jianyang, and Zigong in Sichuan Province, China, was calculated to evaluate the diversity of the *Fusarium* population.

The diversity of *Fusarium* species on intercropped soybean pods was associated with the geographical origin and soybean varieties. As shown in Figure 5, the diversity of *Fusarium* species was distinct for each of the five soybean-growing areas of Sichuan. A total of 28 isolates, identified as *F. fujikuroi*, *F. proliferatum,* and *F. equiseti* were obtained from Chongzhou, 11 isolates of *F. fujikuroi, F. proliferatum*, *F. equiseti,* and *F. incarnatum* from Renshou, 13 isolates of *F. fujikuroi, F. equiseti,* and *F. incarnatum* from Zigong, 14 isolates as *F. fujikuroi* and *F. equiseti* from Jianyang, and 17 isolates of *F. fujikuroi, F. proliferatum,* and *F. graminearum* from Nanchong, respectively. Remarkably, the population of *Fusarium* species was the most diverse in Renshou compared to other areas in Sichuan Province, China. Additionally, *F. fujikuroi* was isolated with a maximum frequency of 78.5% in Jianyang, 76.4% in Nanchong, 61.5% in Zigong, and 48.2% in Chongzhou, while *F. proliferatum* and *F. equiseti* had highest isolation frequency (45.4% and 37.9%) from Renshou and Chongzhou, respectively. Moreover, *F. incarnatum* was obtained with the lowest isolation frequency in Renshou and Zigong. However, *F. graminearum* was isolated only from Nanchong with a low isolation frequency, i.e., 17.64%. Thus, the diversity of the *Fusarium* species showed an association with geographical origin (Figure 5).

**Figure 6:**
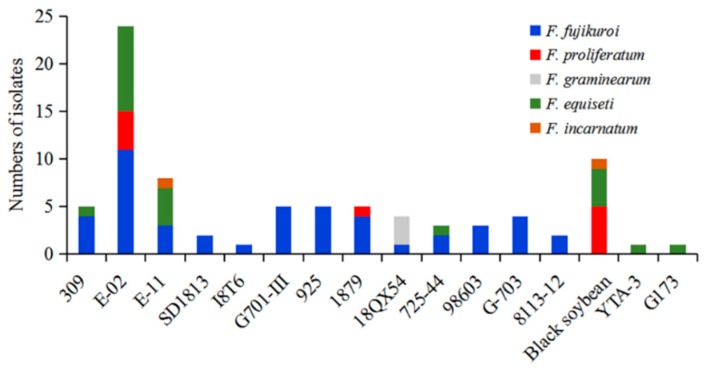
*Fusarium* species isolated from different soybean varieties. Tissue specificity of *Fusarium* species isolated from 16 different varieties of intercropped soybean grown in Sichuan, China.

In the present study, a total of 83 *Fusarium* isolates were recovered from 16 different soybean varieties (Table 1 and Figure 5). *Fusarium fujikuroi* and *F. equiseti* were most aggressive to 81% (13/16 varieties) and 43% (7/16 varieties) of total varieties, respectively, followed by 18% *F. proliferatum* (3/16 varieties), 12% *F. incarnatum* (2/16 varieties), and 6% *F. graminearum* (1/16 varieties) (Figure 6). In our results, *F. fujikuroi* and *F. equiseti* were found to be the most aggressive to soybean E-02, followed by *F. proliferatum*. Interestingly, *F. graminearum* and *F. incarnatum* did not affect the variety of E-02 (Figure 6). Thus, our results showed some specific relationship of the specificity and aggressiveness of *Fusarium* species with different soybean varieties cultivated in Sichuan Province, China.

## 3. Discussion

Many studies have demonstrated that various *Fusarium* species are associated with pod and seed decay, significantly affecting the quality and quantity of soybean [22,23,24,25,40]. In the present study, we identified *F. fujikuroi*, *F. graminearum*, *F. proliferatum*, and *F. incarnatum*-equiseti species complex from diseased soybean pods in intercropped soybean based on the morphological characteristics and phylogenetic analyses of the *EF1-α* and *RPB2* gene sequences. All these species were pathogenic to the seeds of local intercropped soybean cultivar Nandou12, while *F. fujikuroi* was predominantly isolated with the highest level of aggressiveness. We also found that the diversity of the pathogenic *Fusarium* species varied with the geographical origin and soybean varieties. Previous studies demonstrated that a complex of seven *Fusarium* species, i.e., *F. oxysporum*, *F. equiseti*, *F. graminearum*, *F. solani*, *F. commune*, *F. proliferatum*, and *F. avenaceum*, caused soybean root rot [26]. However, the diversity and population of *Fusarium* species on soybean pods were quite distinct in the current study. Moreover, *F. equiseti*, *F. graminearum*, and *F. proliferatum* were found in both soybean diseases (root rot and pod decay) but significantly differed in their aggressiveness, depending upon the distinct soybean organs, thus displaying a certain organ-specificity of *Fusarium* species in soybean. 

It is noteworthy that several *Fusarium* species, such as *F. proliferatum*, *F. graminearum*, *F. fujikuroi,* and *F. equiseti,* have been recognized as pathogens of maize stalk rot [31,41]; in this study, they were also associated with intercropped soybean pods. Among them, *F. graminearum* is the most prevalent pathogen to cause *Fusarium* head blight (FHB) in wheat or barley [42,43], and maize stalk and ear rot [31], and it has recently been isolated from soybean root and seeds in the USA, Argentina, and China [22,24,25,29,44,45]. In our findings, *F. graminearum* significantly inhibited seed germination, reduced seed weight, and caused seed discoloration, indicating that *F. graminearum* causing soybean pod decay might further develop into soybean seeds. This is supported by Barros et al. [21], who reported that *F. graminearum* was abundantly isolated from soybean pods and seeds, indicating a specific penetration ability of *F. graminearum* into seeds. In another study, *F. graminearum* and *F. proliferatum*, which are responsible for ear rot in maize, were reported as the inocula for soybean seedling and root diseases; they also had the ability to reduce soybean seed germination and vigor [45]. In our results, *F. proliferatum* isolated from intercropped soybean pods exhibited lower pathogenicity on the seeds, whereas *F. fujikuroi* showed the most aggressiveness. This result is not in agreement with that of Chang et al. [26]. Previously, *F. incarnatum* and *F. equiseti* often had a close phylogenetic relationship and are classified into a genetic clade [46]. In the current study, these two species were classified into a complex species; they shared one big clade, but in different branches in the phylogenetic trees based on either the *EF1-α* or the *RPB2* gene. In addition, *F. equiseti* reduced seed germination, and caused stunted root growth and partial seed discoloration; this is also reported by Chang et al. [26], who described the effect of *F. equiseti* on seeds germination, seedling growth, and root necrosis. Surprisingly, *F. verticillioides* (syn. *F. moniliforme* Sheldon) causes the most adverse effects on the development of maize seedlings, and adult plants, seeds [23,30,31,41,47,48]; it is also recovered from soybean pods as a pathogen of field pod mold [17,19]. However, in our study, it was not isolated from intercropped soybean pods. With respect to intercropping, *F. verticillioides* might appear saprophytically on soybean pods after maize harvest, but not as a parasite inside the pods. On the other hand, this also might be associated with the fungi isolation methods, i.e., either through tissue isolation after surface-sterilization instead of direct spraying the mildew layer from field pod as described [17]. As the cropping of wheat-maize rotation or wheat-maize/soybean strip intercropping in combination with no-tillage and reduced-tillage is widely practiced in the soybean growing area of southwest China [5], these *Fusarium* species, primarily *F. proliferatum*, *F. graminearum*, *F. fujikuroi*, and *F. equiseti* causing different crop diseases, can exist either saprophytically or parasitically in crop residues from the previous crops, and then produce spores in the subsequent growing season to serve as the primary inoculum source [25,37,45], This leads to an annual cycle-infection on different crops and available growing areas. In addition, *Fusarium* species, as a soil-borne pathogen, can persist in the soil for a long time without any host [20]. Other studies have demonstrated that *Fusarium* species can persist in previous crop residue and contaminate new seeds [49]. A recent survey indicated the high incidence risk of *F. graminearum, F. fujikuroi,* and *F. equiseti* on soybean [26]. 

Besides cropping patterns and conserved agricultural practices, climate conditions and soybean cultivars can significantly affect the infection of *Fusarium* species in host crops [38,50]. It is also reported that a typical karst climate is present in southwest China, especially in Sichuan Province [18]. Low temperature, high rainfall, and low levels of sunshine are favorable to infection, reproduction, colonization, and the spread of *Fusarium* species to different host crops, including wheat, maize, and soybean [26,51]. Delayed harvesting, coupled with unusual weather, causes pod and seed invasion, and pod and seed decay, that severely decrease soybean yield and quality [20]. Recently, a field pod mold caused by *F. veritillioides* was also reported to be associated with local excessive rainfall and low temperatures in Sichuan Province [18]. Chiotta et al. [28] reported that high humidity, higher rainfall, and long-lasting dew during grain ripening were considered to be the most favorable environment for fungal infection, leading to an increase in symptoms of pod blight, high soybean seed infection, and kernel weight reduction. Furthermore, compared with the isolation frequency of *Fusarium* species on different soybean varieties, we found that *F. fujikuroi* and *F. equiseti* were the most aggressive on soybean variety E-02, whereas *F. graminearum* was only isolated from 18QX54 variety, indicating the distinct resistance of soybean to the corresponding *Fusarium* species. Recently, Liu et al. [18] reported a mildew-index-model-based cluster analysis, which was used to evaluate the correlation of the soybean constituents with different organ-specific resistance against field mold; their results demonstrated that proteins, fatty acids, and carbohydrates increased the seed mildew index but decreased the pod mildew index in different soybean genotypes, indicating that the constituents of the soybean pods contributing pod mildew also increased the possibility of the corresponding seed mildew to a certain extent [17,18]. Actually, in order to adopt the specific conditions of light and soil nutrients in maize/soybean strip intercropping, a compact, lodging-resistant soybean genotype is needed [5], but such a variety of soybean has not yet been developed.

Previous literature has reported that pod infection had a direct relationship with seed infection and resulted in weak emergence, stunted plant growth, and yield loss in soybean [40,52]. Seed decay caused by *Phomopsis spp.* can lead to the pod and stem blight [20,40]. Recently, a prediction method of *Phomopsis* seed decay was performed by measuring soybean pod infection [53]. Other studies also confirmed that in the late season, pod and leaf diseases contributed to a significant loss/damage of soybean seeds [54]. These findings indicated that there is a close relationship between pod and seed infection caused by fungi. In our study, the pathogenicity of *Fusarium* species isolated from soybean pods was tested on the seeds; we found that all representative *Fusarium* species infected soybean seeds at different disease levels. *F. fujikuroi* had higher disease severity, poor emergence, and less seed weight, followed by *F. equiseti, F. graminearum, F. proliferatum,* and *F. incarnatum*. It is obvious that pod infection has a direct connection with seed decay. Thus, the findings of the current study may be helpful for regional resistance breeding of soybean and for distinguishing the major and minor pathogens associated with soybean pod decay in Sichuan Province. 

## 4. Materials and Methods 

### 4.1. Sample Collection and Pathogen Isolation

A total of 102 infected soybean pods from 16 different soybean varieties, characterized by rotting, discoloration, and the presence of in white and pink mycelia were collected during the cropping season of 2018 from various fields. These pods were collected from five intercropped soybean-growing areas: Chongzhou (30.6301° N, 103.673° E), Renshou (29.9956° N, 104.1341° E), Nanchong (30.8378° N, 106.1107° E), Jianyang (30.4113° N, 104.502° E), and Zigong (29.339° N, 104.7784° E) in Sichuan Province. After washing the pods with running tap water to remove dust particles, symptomatic pods were excised into 4–8 mm fragments. These fragments were surface-sterilized sequentially with 1% sodium hypochlorite (W/V) for 1 min, 75% ethanol (V/V) for 2 min, rinsed three times in sterilized distilled water, and then air-dried on sterile filter papers under aseptic conditions. The surface-sterilized pods were plated onto potato dextrose agar (PDA, potato 200 g∙L^−1^, glucose anhydrous 10 g∙L^−1^, and agar 15 g∙L^−1^) supplemented with 50 μg∙mL^−1^ streptomycin, and incubated at 25 ± 2 °C in the dark for seven days [26]. Isolates were purified by picking a hyphal tip from the actively-grown colonial margin [55] and preserved on PDA for further identification.

### 4.2. Morphological Characterization

For the morphological identification of these isolates, the cultural characteristics of the colony, mycelium, and colony color were observed on PDA after 7 days of incubation in the dark at 25 ± 2 °C. Colony growth was observed from two days after incubation, and the growth rate of colonies was calculated after seven days’ culture. More than 10 mycelium cakes (5 mm in diameter) from each isolate cultured on PDA were inoculated into 25 mL flasks containing either CMC (7.5 g∙L^−1^ carboxymethyl cellulose sodium, 0.5 g∙L^−1^ yeast extract, 2.5 g∙L^−1^ K_2_HPO_4_, and 0.25 g∙L^−1^ MgSO_4_∙7H_2_O) or PDA liquid medium; macroconidia were incubated in a shaker at 25 ± 2 °C, 120 r·min^−1^ for 5–7 days [26]. Several macroconidial features, including shape, size, and the number of septums, were recorded from 50–100 spores of each species using a compound microscope (Eclipse 80i, Nikon, Japan) [56].

### 4.3. Molecular Identification Based on EF1-α and RPB2 Sequence Analysis

The genomic DNA was extracted from seven-day-old *Fusarium* isolates according to the standard protocols of SP Fungal DNA Kit (Aidlab Biotech, Chengdu, China). The quantity and quality of total extracted DNA was evaluated using a NanoDrop™ 2000 Spectrophotometer (Thermo Scientific, Wilmington, DE, USA). Molecular identification of *Fusarium* species was confirmed by PCR amplification using primer pairs of EF1–728F (5′cat cga gaa gtt cga gaa gg3′) and EF4–986R (5′tac ttg aag gaa ccc tta cc3′) for the partial *EF1-α* gene [57], and RPB2–5f2 (GGGGWGAYCAGAAGAAGGC) and RPB2–7cr (CCCATRGCTTGYTTRCCCAT) for the *RPB2* gene [58]. Total 25 µL of PCR reaction contained genomic DNA 1 μL, each primer 1 μL (10 μM), sterilized water 9.5 μL, and Taq PCR Mastermix 12.5 μL, while genomic DNA was replaced with sterile water as a negative control. Amplification was carried out by S-1000TM Thermal Cycler (Bio-Rad, Foster City, CA, USA) following an initial denaturation at 94 °C for 5 min, 34 threshold cycles of denaturation at 94 °C for 45 s, and annealing of 45 s at 58 °C for *EF1-α* and 55 °C for *RPB2*, respectively, followed by elongation at 72 °C for 2 min, and the final extension at 72 °C for 10 min. The amplified transcript fragments were examined through 1% (w/v) agarose gel electrophoresis; successfully amplified samples were sequenced using an ABI-PRISM3730 automatic sequencer (Applied Biosystems, Foster City, CA, USA). 

The obtained sequences were edited with the BioEdit software v.7.0.5.3 developed by Tom Hall (https://www.filemoro.com/bioedit-free-download/) and an analysis of sequence similarity was conducted with previously-reported *EF1-α* and *RPB2* sequences from Fusarium MLST (http://www.wi.knaw.nl/Fusarium/Biolomics.aspx) and FUSARIUM-ID (http://isolate.fusariumdb.org/guide.php) database [59]. All obtained and reference sequences from NCBI were aligned using Clustal X 1.83 developed by SGI (http://www.sgi.com/industries/sciences/chembio/resources/clustalw/parallel_clustalw.html). A phylogenetic tree was constructed by MEGA 7.0.26 using the maximum-likelihood method based on the Tamura–Nei model [60]. One thousand bootstrap replicates were used for clade support, and alignment gaps were removed. Prestigious sequence data were submitted in the alignment in TreeBASE (www.treebase.org) and in GenBank. 

### 4.4. Pathogenicity Tests

Koch’s postulate was used to test the pathogenicity of the identified *Fusarium* species by inoculation of spore suspension. The impact of three representative isolates of *Fusarium* species (*F.*, *F. proliferatum*, *F. equiseti*, and *F. graminearum*) and two isolates of *F. incarnatum* was checked on asymptomatic seeds of soybean cultivar “Nandou12”. The seed soaking method was used according to [61] with minor modifications in detail. A spore suspension was prepared by transferring about five mycelium disks of *Fusarium* isolates into 20 mL of CMC or PDA medium and incubating at 150 r·min^−1^ and 25 °C on an orbital shaker for 7 days. The final concentration of 1 × 10^5^ spore mL^−1^ was adjusted and used for inoculation. Soybean seeds were surface sterilized with 1% sodium hypochlorite solution for 1 min, rinsed three times in sterile distilled water, and air-dried on sterile filter paper under aseptic conditions. For each isolate, three plates were prepared with 15 seeds each, and three independent replicates were conducted. Sterilized seeds were dipped in the spore suspension for 5 min, while seeds dipped in sterile distilled water were used as a negative control and incubated at 25 ± 2 °C in the dark with 70% relative humidity. After seven days of inoculation, the symptoms and disease incidence were recorded. For the disease severity index (*DSI*), seeds were assigned a disease severity grade [62] with minor modifications: 0 = healthy seeds germination, 1 = delayed growth with negligible or no discoloration, 2 = germination with isolated lesions, 3 = developed with the merged lesion, and 4 = colonized seeds with no germination. The *DSI* was calculated according to the formula below. The percentage of the mycelium-covering area (*PMC*) on the seed surface was also evaluated. Additionally, the germination rate, seed weight and root length of the soybeans were documented. For statistical analysis, Dunnett’s test was used by SPSS to determine significant differences.

$$DSI=\frac{\sum (severity\;rating\times seeds\;per\;rating)}{\left(total\;seeds\times highest\;severity\;rating\right)}\times 100$$

## 5. Conclusions

It is concluded that *F. fujikuroi*, *F. proliferatum*, *F. graminearum*, and *F. incarnatum-equiseti species* complex were successfully isolated and identified from the intercropped soybean pods. The diversity of *Fusarium* species was closely related to geographical origin and soybean variety, and this may be considered in the screening of new soybean cultivars that are suitable for the maize/soybean strip intercropping according to local climatic conditions and agricultural practices. Climatic conditions probably played a significant role in the incidence and epidemics of soybean pod decay caused by different *Fusarium* species. So far, the chemical and molecular responses of soybean against these *Fusarium* species are still not clear, especially concerning *F. graminearum* under the favorable conditions. In the future, an interaction of the dominant *Fusarium* species with different hosts and the epidemiology, etiology, and management of soybean pod decay will be focused upon. The findings of the current study provide useful information for the management of *Fusarium*-associated soybean pods and seed diseases in maize/soybean strip intercropping in southwest China.

## Figures and Tables

**Table 1:** Information of *Fusarium* species isolated from soybean pods collected from different growing areas of maize/soybean strip intercropping in Sichuan, China.

Isolates	Soybean Cultivars	Sampling Location	Suggested Identification	GenBank Accession Number	
				*EF1-α*	*RPB2*
SP01	309	Chongzhou, Sichuan	*F. fujikuroi*	MK611857	MN625641
SP02	309	Chongzhou, Sichuan	*F. fujikuroi*	MK611865	MN625642
SP03	309	Chongzhou, Sichuan	*F. fujikuroi*	MK611868	MN625643
SP04	309	Changzhou, Sichuan	*F. fujikuroi*	MK611861	MN625644
SP004	309	Changzhou, Sichuan	*F. equiseti*	MK611911	MN625626
SP10	E-02	Chongzhou, Sichuan	*F. fujikuroi*	MK611843	MN625645
SP010	E-02	Chongzhou, Sichuan	*F. fujikuroi*	MK611871	MN625669
SP13	E-02	Chongzhou, Sichuan	*F. fujikuroi*	MK611874	MN625672
SP14	E-02	Chongzhou, Sichuan	*F. fujikuroi*	MK611858	MN625659
SP19	E-02	Chongzhou, Sichuan	*F. fujikuroi*	MK611844	MN625646
SP20	E-02	Chongzhou, Sichuan	*F. fujikuroi*	MK611869	MN625667
SP21	E-02	Chongzhou, Sichuan	*F. fujikuroi*	MK611863	MN625663
SP28	E-02	Chongzhou, Sichuan	*F. fujikuroi*	MK611864	MN625664
SP32	E-02	Chongzhou, Sichuan	*F. fujikuroi*	MK611870	MN625668
SP07	E-02	Chongzhou, Sichuan	*F. equiseti*	MK611917	MN625632
SP08	E-02	Chongzhou, Sichuan	*F. equiseti*	MK611923	MN625638
SP12	E-02	Chongzhou, Sichuan	*F. equiseti*	MK611921	MN625636
SP16	E-02	Chongzhou, Sichuan	*F. equiseti*	MK611913	MN625628
SP23	E-02	Chongzhou, Sichuan	*F. equiseti*	MK611912	MN625627
SP30	E-02	Chongzhou, Sichuan	*F. equiseti*	MK611919	MN625634
SP31	E-02	Chongzhou, Sichuan	*F. equiseti*	MK611922	MN625637
SP24	E-02	Chongzhou, Sichuan	*F. proliferatum*	MK611898	MN625696
SP27	E-02	Chongzhou, Sichuan	*F. proliferatum*	MK611892	MN625690
SP027	E-02	Chongzhou, Sichuan	*F. proliferatum*	MK611889	MN625687
SP29	E-02	Chongzhou, Sichuan	*F. proliferatum*	MK611890	MN625688
SP34	Black soybean	Chongzhou, Sichuan	*F. equiseti*	MK611920	MN625635
SP35	Black soybean	Chongzhou, Sichuan	*F. equiseti*	MK611916	MN625631
SP36	Black soybean	Chongzhou, Sichuan	*F. equiseti*	MK611914	MN625629
SP43	Black soybean	Renshou, Sichuan	*F. equiseti*	MK611910	MN625625
SP37	Black soybean	Renshou, Sichuan	*F. proliferatum*	MK611897	MN625695
037	Black soybean	Renshou, Sichuan	*F. proliferatum*	MK611894	MN625692
SP39	Black soybean	Renshou, Sichuan	*F. proliferatum*	MK611893	MN625691
SP039	Black soybean	Renshou, Sichuan	*F. proliferatum*	MK611891	MN625689
SP40	Black soybean	Renshou, Sichuan	*F. proliferatum*	MK611896	MN625694
SP44	Black soybean	Renshou, Sichuan	*F. incarnatum*	MK611902	MN625700
SP52	E-11	Zigong, Sichuan	*F. fujikuroi*	MK611880	MN625678
SP53	E-11	Jianyang, Sichuan	*F. fujikuroi*	MK611873	MN625671
SP053	E-11	Jianyang, Sichuan	*F. fujikuroi*	MK611866	MN625665
SP47	E-11	Renshou, Sichuan	*F. equiseti*	MK611907	MN625622
SP48	E-11	Zigong, Sichuan	*F. equiseti*	MK611908	MN625623
SP49	E-11	Zigong, Sichuan	*F. equiseti*	MK611909	MN625624
SP50	E-11	Zigong, Sichuan	*F. equiseti*	MK611905	MN625620
SP51	E-11	Zigong, Sichuan	*F. incarnatum*	MK611903	MN625701
SP58	E-02	Jianyang, Sichuan	*F. fujikuroi*	MK611879	MN625677
SP61	E-02	Jianyang, Sichuan	*F. fujikuroi*	MK611862	MN625662
SP60	E-02	Jianyang, Sichuan	*F. equiseti*	MK611906	MN625621
SP62	E-02	Jianyang, Sichuan	*F. equiseti*	MK611918	MN625633
SP70	YT-A3	Chongzhou, Sichuan	*F. equiseti*	MK611915	MN625630
SP74	SD18113	Renshou, Sichuan	*F. fujikuroi*	MK611854	MN625656
SP75	SD18113	Renshou, Sichuan	*F. fujikuroi*	MK611867	MN625666
SP81	I8T6	Renshou, Sichuan	*F. fujikuroi*	MK611852	MN625654
SP84	G701-III	Zigong, Sichuan	*F. fujikuroi*	MK611842	MN625640
SP85	G701-III	Zigong, Sichuan	*F. fujikuroi*	MK611859	MN625660
SP86	925	Zigong, Sichuan	*F. fujikuroi*	MK611882	MN625680
SP87	925	Zigong, Sichuan	*F. fujikuroi*	MK611848	MN625650
SP89	701-III	Zigong, Sichuan	*F. fujikuroi*	MK611885	MN625683
SP90	701-III	Zigong, Sichuan	*F. fujikuroi*	MK611855	MN625657
SP91	701-III	Zigong, Sichuan	*F. fujikuroi*	MK611888	MN625686
SP94	1879	Nanchong, Sichuan	*F. fujikuroi*	MK611881	MN625679
SP95	1879	Nanchong, Sichuan	*F. fujikuroi*	MK611877	MN625675
SP97	1879	Nanchong, Sichuan	*F. fujikuroi*	MK611860	MN625661
SP98	1879	Nanchong, Sichuan	*F. fujikuroi*	MK611887	MN625685
SP097	1879	Nanchong, Sichuan	*F. proliferatum*	MK611895	MN625693
SP107	18QX54	Nanchong, Sichuan	*F. fujikuroi*	MK611846	MN625648
SP99	18QX54	Nanchong, Sichuan	*F. graminearum*	MK611899	MN625697
SP100	18QX54	Nanchong, Sichuan	*F. graminearum*	MK611901	MN625698
SP102	18QX54	Nanchong, Sichuan	*F. graminearum*	MK611900	MN625699
SP110	725–44	Nanchong, Sichuan	*F. fujikuroi*	MK611872	MN625670
0110	725–44	Nanchong, Sichuan	*F. fujikuroi*	MK611851	MN625653
SP111	725–44	Nanchong, Sichuan	*F. equiseti*	MK611924	MN625638
SP113	925	Nanchong, Sichuan	*F. fujikuroi*	MK611878	MN625676
SP114	925	Nanchong, Sichuan	*F. fujikuroi*	MK611875	MN625673
SP115	925	Nanchong, Sichuan	*F. fujikuroi*	MK611853	MN625655
SP117	98603	Nanchong, Sichuan	*F. fujikuroi*	MK611883	MN625681
SP118	98603	Nanchong, Sichuan	*F. fujikuroi*	MK611876	MN625674
SP119	98603	Jianyang, Sichuan	*F. fujikuroi*	MK611850	MN625652
SP120	G-703	Jianyang, Sichuan	*F. fujikuroi*	MK611845	MN625647
SP121	G-703	Jianyang, Sichuan	*F. fujikuroi*	MK611884	MN625682
SP122	G-703	Jianyang, Sichuan	*F. fujikuroi*	MK611847	MN625649
SP123	G-703	Jianyang, Sichuan	*F. fujikuroi*	MK611849	MN625651
SP125	G173–12	Jianyang, Sichuan	*F. equiseti*	MK611904	MN625619
SP127	8113–12	Jianyang, Sichuan	*F. fujikuroi*	MK611886	MN625684
SP129	8113–12	Jianyang, Sichuan	*F. fujikuroi*	MK611856	MN625658

Note: *Fusarium* species were identified based on the homology analysis of partial sequences of the translation elongation factor 1-α (*EF1-α*) and RNA polymerase II second largest subunit (*RPB2*) genes on *Fusarium* MLST and FUSARIUM-ID database.

**Table 2:** Morphological characteristics of *Fusarium* species isolated from intercropped soybean pods in different growing areas in Sichuan, China.

*Fusarium* Species	Growth Rate (cm/day)	Colony Characterization	Macroconidia			
			Septum	Length	Width	Shape
*F. fujikuroi*	4.08 ± 0.26 c	Pale grey color (front), pale yellowish color (back)	3–5	40.38 ± 2.91 b 44.78–36.02	2.48 ± 0.23 c 3.17–2.17	Falcate
*F. proliferatum*	3.30 ± 0.02 d	Pale grey color (front), pale grey (back)	3–4	39.65 ± 7.88 b 50.33–30.59	3.69 ± 0.90 b 5.41–1.86	Falcate, fusiform
*F. graminearum*	5.34 ± 0.22 a*	White yellowish (front), purple (back)	4–6	45.90 ± 4.40 a* 48.81–31.43	4.42 ± 0.75 a* 5.14–2.32	Falcate
*F. equiseti*	4.62 ± 0.44 b	Pale grey color (front), ginger yellowish (back)	3–4	29.61 ± 3.84 e 34.46–21.47	3.12 ± 0.43 c 4.05–2.01	Falcate
*F. incarnatum*	4.92 ± 0.19 ab	Pale grey color(front), yellowish color (back)	3–4	37.46 ± 4.98 c 47.02–33.57	4.03 ± 1.06 b 5.89–1.86	Falcate

Note: Data were the mean from three independent replicates. *: Different lowercase in the same column shows a significant difference at the level of *p* = 0.05, according to Duncan’s multiple range test.

**Table 3:** Disease occurrence and growth parameters of soybean after inoculation with the representative isolates of *Fusarium* species isolated from intercropped soybean pods.

Isolate name	Isolate ID	Percentage of Mycelium Covering (PMC)	Seed Weight (g)	Root Length (CM)	Germination Rate (%)	Disease Severity Index (DSI)
control	C01	0 g*	0.91 ± 0.05 a*	3.30 ± 0.17 a*	100 ± 0 a*	0 g*
	C02	0 g	0.84 ± 0.09 ab	2.93 ± 0.35 ab	100 ± 0 a	0 g
	C03	0 g	0.82 ± 0.11 abc	3.21 ± 0.63 a	100 ± 0 a	0 g
*F. fujikuroi*	SP95	82.00 ± 19.05 ab	0.40 ± 0.01 j	1.04 ± 1.15 efgh	57.33 ± 15.01 d	70.50 ± 7.77 a
	SP14	82.10 ± 13.74 ab	0.42 ± 0.01 j	1.15 ± 0.63 c	55.00 ± 25.35 d	70.04 ± 2.42 a
	SP59	90.88 ± 3.66 a	0.47 ± 0.02 ij	1.01 ± 0.75 fghi	57.66 ± 6.80 d	71.66 ± 7.07 a
*F. equiseti*	SP30	59.66 ± 13.50 cd	0.50 ± 0.02 hij	0.99 ± 0.33 ghi	77.33 ± 15.01 bcd	64.15 ± 5.89 bc
	SP31	70.99 ± 20.18 bc	0.50 ± 0 hij	0.61 ± 0.42 i	79.66 ± 17.38 abc	60.00 ± 0 bcd
	SP08	71.10 ± 3.80 abc	0.61 ± 0.01 fghi	0.72 ± 0.40 hi	79.66 ± 11.54 abc	60.83 ± 3.53 ab
*F. graminearum*	SP99	56.00 ± 20.00 cde	0.59 ± 0.03 hij	1.78 ± 0.05 defg	76.00 ± 10 bcd	55.83 ± 5.89 bcd
	SP100	52.22 ± 10.18 cde	0.62 ± 0.04 fghi	1.75 ± 0.32 defg	66.63 ± 23.71 cd	55.83 ± 3.53 bcd
	SP102	57.55 ± 14.14 cd	0.65 ± 0.01 efg	1.47 ± 0.34 efghi	77.33 ± 7.50 bcd	59.99 ± 2.35 bcd
*F. proliferatum*	SP29	28.44 ± 4.23 ef	0.68 ± 0.05 defg	2.54 ± 0.14 defgh	77.33 ± 14.01 bcd	45.83 ± 8.24 ef
	SP24	35.33 ± 10.47 ef	0.65 ± 0.03 efg	2.07 ± 0.78 bcde	79.66 ± 17.38 abc	43.33 ± 0 ef
	SP37	37.55 ± 14.14 e	0.70 ± 0.07 def	1.95 ± 0.13 cdef	88.33 ± 4.04 ab	49.16 ± 1.18 de
*F. incarnatum*	SP44	24.00 ± 3.46 f	0.73 ± 0.05 cde	2.71 ± 0.11 abc	88.33 ± 4.04 ab	35..83 ± 8.24 f
	SP51	26.44 ± 6.3 f	0.76 ± 0.08 bcd	1.84 ± 0.17 abc	81.66 ± 14.01 abc	40.44 ± 2.11 f

Note: Data were the mean from three independent replicates. *: Different lowercase in the same column shows a significant difference at the level of *p* = 0.05, according to Duncan’s multiple range test.

## Supplementary Materials

The following are available online at https://www.mdpi.com/2076-0817/8/4/245/s1, Figure S1: Typical colonies and macroconidia of *Fusarium* isolate from soybean pods in the maize/soybean strip intercropping, Table S1: Reference sequences of *EF1-α* and *RPB2* gene from Genebank used for the phylogenetic analysis of *Fusarium* species associated with the intercropped soybean pods.

## References

[1] Yu H., Liu R., Hu Y., Xu B. (2017). Flavor profiles of soymilk processed with four different processing technologies and 26 soybean cultivars grown in China. Int. J. Food Prop..

[2] Zhang Q., Wang C., Li B., Li L., Lin D., Chen H., Liu Y., Li S., Qin W., Liu J. (2018). Research progress in tofu processing: From raw materials to processing conditions. Crit. Rev. Food Sci. Nutr..

[3] Lee G.A., Crawford G.W., Liu L., Sasaki Y., Chen X. (2011). Archaeological soybean (*Glycine max*) in East Asia: Does size matter?. PLoS ONE.

[4] Zhang F., Chen X., Vitousek P. (2013). Chinese agriculture: An experiment for the world. Nature.

[5] Du J., Han T., Gai J., Yong T., Sun X., Wang X., Yang F., Liu J., Shu K., Liu W. (2018). Maize-soybean strip intercropping: Achieved a balance between high productivity and sustainability. J. Integr. Agric..

[6] Su B., Liu X., Cui L., Xiang B., Yang W. (2018). Suppression of Weeds and Increases in Food Production in Higher Crop Diversity Planting Arrangements: A Case Study of Relay Intercropping. Crop Sci..

[7] Liu X., Rahman T., Song C., Su B., Yang F., Yong T., Wu Y., Zhang C., Yang W. (2017). Changes in light environment, morphology, growth and yield of soybean in maize-soybean intercropping systems. Field Crop. Res..

[8] Liu X., Rahman T., Song C., Yang F., Su B., Cui L., Bu W., Yang W. (2018). Relationships among light distribution, radiation use efficiency and land equivalent ratio in maize-soybean strip intercropping. Field Crop. Res..

[9] Yong T., Ping C., Qian D., Qing D., Feng Y., WANG X., LIU W., YANG W. (2018). Optimized nitrogen application methods to improve nitrogen use efficiency and nodule nitrogen fixation in a maize-soybean relay intercropping system. J. Integr. Agric..

[10] Raza M.A., Khalid M.H.B., Zhang X., Feng L.Y., Khan I., Hassan M.J., Ahmed M., Ansar M., Chen Y.K., Fan Y.F. (2019). Effect of planting patterns on yield, nutrient accumulation and distribution in maize and soybean under relay intercropping systems. Sci. Rep..

[11] Raza M.A., Feng L.Y., van der Werf W., Cai G.R., Khalid M.H.B., Iqbal N., Hassan M.J., Meraj T.A., Naeem M., Khan I. (2019). Narrow-wide-row planting pattern increases the radiation use efficiency and seed yield of intercrop species in relay-intercropping system. Food Energy Secur..

[12] Chen P., Song C., Liu X., Zhou L., Yang H., Zhang X., Zhou Y., Du Q., Pang T., Fu Z. (2019). Yield advantage and nitrogen fate in an additive maize-soybean relay intercropping system. Sci. Total Environ..

[13] Qin A., Huang G., Chai Q., Yu A., Huang P. (2013). Grain yield and soil respiratory response to intercropping systems on arid land. Field Crop. Res..

[14] Yang F., Wang X., Liao D., Lu F., Gao R., Liu W., Yong T., Wu X., Du J., Liu J. (2015). Yield response to different planting geometries in maize-soybean relay strip intercropping systems. Agron. J..

[15] Raza M.A., Feng L.Y., Khalid M.H., Iqbal N., Meraj T.A., Hassan M.J., Ahmed S., Chen Y.K., Feng Y., Wenyu Y. (2019). Optimum leaf excision increases the biomass accumulation and seed yield of maize plants under different planting patterns. Ann. Appl. Biol..

[16] Keesstra S., Nunes J., Novara A., Finger D., Avelar D., Kalantari Z., Cerdà A. (2018). The superior effect of nature based solutions in land management for enhancing ecosystem services. Sci. Total Environ..

[17] Liu B., Chen C., Lian Y., Chen J., Chen X. (2015). Long-term change of wet and dry climatic conditions in the southwest karst area of China. Glob. Planet. Chang..

[18] Liu J., Deng J., Zhang K., Wu H., Yang C., Zhang X., Du J., Shu K., Yang W. (2016). Pod mildew on soybeans can mitigate the damage to the seed arising from field mold at harvest time. J. Agric. Food Chem..

[19] Liu J., Deng J., Yang C., Huang N., Chang X., Zhang J., Yang F., Liu W., Wang X., Yong T. (2017). Fungal diversity in field mold-damaged soybean fruits and pathogenicity identification based on high-throughput rDNA sequencing. Front. Microbiol..

[20] Roy K., Ratnayake S., McLean K. (1997). Colonization of weeds by Phomopsis longicolla. Can. J. Plant Pathol..

[21] Barros G., Zanon M.A., Abod A., Oviedo M., Ramirez M., Reynoso M., Torres A., Chulze S. (2012). Natural deoxynivalenol occurrence and genotype and chemotype determination of a field population of the *Fusarium graminearum* complex associated with soybean in Argentina. Food Addit. Contam. Part A.

[22] Barros G.G., Zanon M.S.A., Chiotta M.L., Reynoso M.M., Scandiani M.M., Chulze S.N. (2014). Pathogenicity of phylogenetic species in the *Fusarium graminearum* complex on soybean seedlings in Argentina. Eur. J. Plant Pathol..

[23] Garcia D., Barros G., Chulze S., Ramos A.J., Sanchis V., Marín S. (2012). Impact of cycling temperatures on *Fusarium verticillioides* and *Fusarium graminearum* growth and mycotoxins production in soybean. J. Sci. Food Agric..

[24] Ellis M., Broders K., Paul P., Dorrance A. (2011). Infection of soybean seed by *Fusarium graminearum* and effect of seed treatments on disease under controlled conditions. Plant Dis..

[25] Arias M.M.D., Leandro L.F., Munkvold G.P. (2013). Aggressiveness of *Fusarium* species and impact of root infection on growth and yield of soybeans. Phytopathology.

[26] Chang X., Dai H., Wang D., Zhou H., He W., Fu Y., Ibrahim F., Zhou Y., Gong G., Shang J. (2018). Identification of *Fusarium* species associated with soybean root rot in Sichuan Province, China. Eur. J. Plant Pathol..

[27] Zhang J., Xue A., Cober E., Morrison M., Zhang H., Zhang S., Gregorich E. (2013). Prevalence, pathogenicity and cultivar resistance of *Fusarium* and *Rhizoctonia* species causing soybean root rot. Can. J. Plant Sci..

[28] Chiotta M.L., Alaniz Zanon M.S., Palazzini J.M., Scandiani M.M., Formento A.N., Barros G.G., Chulze S.N. (2016). Pathogenicity of *Fusarium graminearum* and *F. meridionale* on soybean pod blight and trichothecene accumulation. Plant Pathol..

[29] Pioli R., Mozzoni L., Morandi E., Menard M. (2004). Disease Notes. Plant Dis..

[30] Ferrigo D., Raiola A., Causin R. (2016). Fusarium toxins in cereals: Occurrence, legislation, factors promoting the appearance and their management. Molecules.

[31] Miedaner T., Bolduan C., Melchinger A. (2010). Aggressiveness and mycotoxin production of eight isolates each of *Fusarium graminearum* and *Fusarium verticillioides* for ear rot on susceptible and resistant early maize inbred lines. Eur. J. Plant Pathol..

[32] Munkvold G.P. (2003). Epidemiology of *Fusarium* diseases and their mycotoxins in maize ears. Eur. J. Plant Pathol..

[33] Pestka J.J. (2010). Deoxynivalenol: Mechanisms of action, human exposure, and toxicological relevance. Arch. Toxicol..

[34] Nelson B.D., Hansen J.M., Windels C.E., Helms T.C. (1997). Reaction of soybean cultivars to isolates of *Fusarium solani* from the Red River Valley. Plant Dis..

[35] Zhang J., Xue A., Zhang H., Nagasawa A., Tambong J. (2010). Response of soybean cultivars to root rot caused by *Fusarium* species. Can. J. Plant Sci..

[36] Becher R., Hettwer U., Karlovsky P., Deising H.B., Wirsel S.G. (2010). Adaptation of *Fusarium graminearum* to tebuconazole yielded descendants diverging for levels of fitness, fungicide resistance, virulence, and mycotoxin production. Phytopathology.

[37] Chang K., Conner R., Hwang S., Ahmed H., McLaren D., Gossen B., Turnbull G. (2014). Effects of seed treatments and inoculum density of *Fusarium avenaceum* and *Rhizoctonia solani* on seedling blight and root rot of faba bean. Can. J. Plant Sci..

[38] West J.S., Holdgate S., Townsend J.A., Edwards S.G., Jennings P., Fitt B.D. (2012). Impacts of changing climate and agronomic factors on fusarium ear blight of wheat in the UK. Fungal Ecol..

[39] Wei W., Xu Y., Zhu L., Zhang S., Li S. (2014). Impact of long-term continuous cropping on the *Fusarium* population in soybean rhizosphere. Yingyong Shengtai Xuebao.

[40] Roy K., Baird R., Abney T. (2001). A review of soybean (*Glycine max*) seed, pod, and flower mycofloras in North America, with methods and a key for identification of selected fungi. Mycopathologia.

[41] Machado J.D.C., Machado A.Q., Pozza E.A., Machado C.F., Zancan W.L.A. (2013). Inoculum potential of *Fusarium verticillioides* and performance of maize seeds. Trop. Plant Pathol..

[42] Sella L., Gazzetti K., Castiglioni C., Schäfer W., Favaron F. (2014). *Fusarium graminearum* possesses virulence factors common to *Fusarium* head blight of wheat and seedling rot of soybean but differing in their impact on disease severity. Phytopathology.

[43] Zhang H., Van der Lee T., Waalwijk C., Chen W., Xu J., Xu J., Zhang Y., Feng J. (2012). Population analysis of the *Fusarium graminearum species complex* from wheat in China show a shift to more aggressive isolates. PLoS ONE.

[44] Alvarez C.L., Azcarate M.P., Pinto V.F. (2009). Toxigenic potential of *Fusarium graminearum sensu stricto* isolates from wheat in Argentina. Int. J. Food Microbiol..

[45] Díaz Arias M., Munkvold G., Leandro L. (2011). First report of *Fusarium proliferatum* causing root rot on soybean (*Glycine max*) in the United States. Plant Dis..

[46] Watanabe M., Yonezawa T., Lee K.-I., Kumagai S., Sugita-Konishi Y., Goto K., Hara-Kudo Y. (2011). Molecular phylogeny of the higher and lower taxonomy of the *Fusarium* genus and differences in the evolutionary histories of multiple genes. Bmc Evol. Biol..

[47] Pedrozo R., Little C.R. (2017). *Fusarium verticillioides* inoculum potential influences soybean seed quality. Eur. J. Plant Pathol..

[48] Pedrozo R., Little C. (2015). The interesting case of soybean seedborne *Fusarium spp.*: From identity to pathogenicity. Phytopathology.

[49] Osborne L.E., Stein J.M. (2007). Epidemiology of *Fusarium* head blight on small-grain cereals. Int. J. Food Microbiol..

[50] Backhouse D. (2014). Global distribution of *Fusarium graminearum*, *F. asiaticum* and *F. boothii* from wheat in relation to climate. Eur. J. Plant Pathol..

[51] Doohan F., Brennan J., Cooke B. (2003). Influence of climatic factors on *Fusarium* species pathogenic to cereals. Epidemiology of Mycotoxin Producing Fungi.

[52] Mengistu A., Castlebury L., Smith R., Ray J., Bellaloui N. (2009). Seasonal progress of *Phomopsis longicolla* infection on soybean plant parts and its relationship to seed quality. Plant Dis..

[53] McGee D. (1986). Prediction of Phomopsis seed decay by measuring soybean pod infection. Plant Dis..

[54] Jeschke M.R., Stoltenberg D.E., Kegode G.O., Sprague C.L., Knezevic S.Z., Hock S.M., Johnson G.A. (2011). Predicted soybean yield loss as affected by emergence time of mixed-species weed communities. Weed Sci..

[55] Zhou Y., Gong G., Cui Y., Zhang D., Chang X., Hu R., Liu N., Sun X. (2015). Identification of *Botryosphaeriaceae* species causing kiwifruit rot in Sichuan Province, China. Plant Dis..

[56] Leslie J.F., Summerell B.A. (2008). The Fusarium Laboratory Manual.

[57] Chala A., Degefu T., Brurberg M.B. (2019). Phylogenetically diverse *Fusarium* species associated with sorghum (*Sorghum Bicolor* L. Moench) and finger Millet (*Eleusine Coracana* L. Garten) grains from Ethiopia. Diversity.

[58] Laurence M., Walsh J., Shuttleworth L., Robinson D., Johansen R., Petrovic T., Vu T., Burgess L., Summerell B., Liew E. (2016). Six novel species of *Fusarium* from natural ecosystems in Australia. Fungal Divers..

[59] O’Donnell K., Humber A.R., Geiser D.M., Kang S., Park B., Robert V.A., Crous P.W., Johnston R.P., Aoki T., Rooney P.A. (2012). Phylogenetic diversity of insecticolous fusaria inferred from multilocus DNA sequence data and their molecular identification via FUSARIUM-ID and Fusarium MLST. Mycologia.

[60] Kumar S., Stecher G., Tamura K. (2016). MEGA7: Molecular evolutionary genetics analysis version 7.0 for bigger datasets. Mol. Biol. Evol..

[61] Gao X., Wu M., Xu R., Wang X., Pan R., Kim H., Liao H. (2014). Root interactions in a maize/soybean intercropping system control soybean soil-borne disease, red crown rot. PLoS ONE.

[62] Alejandro Rojas J., Jacobs J.L., Napieralski S., Karaj B., Bradley C.A., Chase T., Esker P.D., Giesler L.J., Jardine D.J., Malvick D.K. (2016). Oomycete species associated with soybean seedlings in North America—Part I: Identification and pathogenicity characterization. Phytopathology.

