# Supplementary material for: Characterization and Pathogenicity of Fusarium Species Associated with Soybean Pods in Maize/Soybean Strip Intercropping
Source: Pathogens. 2019 Nov 19;8(4):245. doi: 10.3390/pathogens8040245 (PMC6963259; doi:10.3390/pathogens8040245)
Supplement: Supplementary file 1 [file pathogens-08-00245-s001.zip › Supplementary Materials/Figure S1.docx]

***Pathogens***

**Characterization and pathogenicity of *Fusarium* species associated with soybean pods in the maize/soybean strip intercropping**

Muhammd Naeem^1^, Hongju Li^1^, Li Yan^1^, Muhammad A. Raza^1^, Guoshu Gong^1^, Huabao Chen^1^, Chunping Yang^1^, Min Zhang^1^, Jing Shang^1^, Taiguo Liu^2^, Wanquan Chen^2^, Muhammad Faheem Abbas^3^, Gulshan Irshad^3^, Muhammed I. Khaskheli^4^, Wenyu Yang1, Xiaoli Chang^1,2,*^.

^1^College of Agronomy, Sichuan Agricultural University, Chengdu, 611130, Sichuan Province, P.R. China

^2^State Key Laboratory for Biology of Plant Diseases and Insect Pests, Institute of Plant Protection, Chinese Academy of Agricultural Sciences, Beijing, 100193, P.R. China

^3^Department of Plant Pathology, PMAS Arid Agriculture University, Rawalpindi, 46000, Pakistan

^4^Department of Plant Protection, Faculty of Crop Protection, Sindh Agriculture University, Tandojam, 70060, Pakistan

^*^The corresponding author: [xl_changkit@126.com](mailto:xl_changkit@126.com); Tel.: +86 (0)28 86290870; Fax: +86 (0)28 86290872.


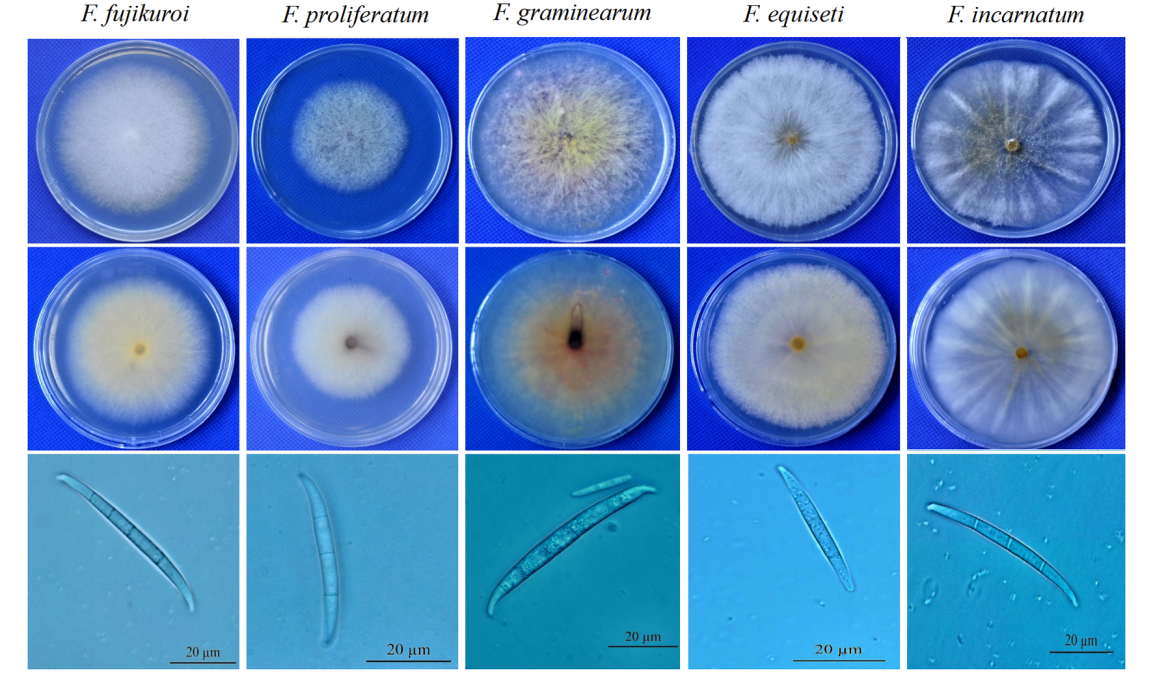


**Figure S1.** Typical colonies and macroconidia of *Fusarium* isolates from soybean pods in the maize/soybean strip intercropping

Typical colonies of *Fusarium* isolates were observed after 5 days grown in PDA and macroconidia (down line) after 5 days grown in either Czapek–Dox or PDA medium. Isolates included *F. fujikuroi* (SP39), *F. proliferatum* (SP37), *F. graminearum* (SP100), *F. equiseti* (SP30), and *F. incarnatum* (SP51). Scale bar=20 μm.
