# Supplementary material for: Characterization and Pathogenicity of Fusarium Species Associated with Soybean Pods in Maize/Soybean Strip Intercropping
Source: Pathogens. 2019 Nov 19;8(4):245. doi: 10.3390/pathogens8040245 (PMC6963259; doi:10.3390/pathogens8040245)
Supplement: Supplementary file 1 [file pathogens-08-00245-s001.zip › Supplementary Materials/Table S1.docx]

***Pathogens***

**Characterization and pathogenicity of *Fusarium* species associated with soybean pods in the maize/soybean strip intercropping**

Muhammd Naeem^1^, Hongju Li^1^, Li Yan^1^, Muhammad A. Raza^1^, Guoshu Gong^1^, Huabao Chen^1^, Chunping Yang^1^, Min Zhang^1^, Jing Shang^1^, Taiguo Liu^2^, Wanquan Chen^2^, Muhammad Faheem Abbas^3^, Gulshan Irshad^3^, Muhammed I. Khaskheli^4^, Wenyu Yang1, Xiaoli Chang^1,2,*^.

^1^College of Agronomy, Sichuan Agricultural University, Chengdu, 611130, Sichuan Province, P.R. China

^2^State Key Laboratory for Biology of Plant Diseases and Insect Pests, Institute of Plant Protection, Chinese Academy of Agricultural Sciences, Beijing, 100193, P.R. China

^3^Department of Plant Pathology, PMAS Arid Agriculture University, Rawalpindi, 46000, Pakistan

^4^Department of Plant Protection, Faculty of Crop Protection, Sindh Agriculture University, Tandojam, 70060, Pakistan

^*^The corresponding author: [xl_changkit@126.com](mailto:xl_changkit@126.com); Tel.: +86 (0)28 86290870; Fax: +86 (0)28 86290872.

**Table S1.** Reference sequences of EF1-α gene from Genebank used for the phylogenetic analysis of *Fusarium* species associated with the intercropped soybean pods.

| ***Fusarium* isolates** | ***Fusarium* species** | **GeneBank accession numbers (EF1-α)** |
| --- | --- | --- |
| BJ-1 | *Fusarium fujikuroi* | MH263736 |
| D71 | *F. fujikuroi* | KX922702 |
| BJY2 | 1. *Fujikuroi* | MF996498 |
| CBS 262.54 | *F. fujikuroi* | KR071744 |
| F268 | *F. fujikuroi* | KP009966 |
| XJSF | *F. proliferatum* | MF083156 |
| XJSFB | *F. proliferatum* | MG557986 |
| D66 | *F. proliferatum* | KX966216 |
| B12 | *Fusarium graminearum* | KX269097 |
| 17XY13-4 | *F. graminearum* | MG231125 |
| Xm2016074-2 | *Fusarium incarnatum* | MG882013 |
| DG1301 | *F. incarnatum* | KT313002 |
| Indo140 | *F. equiseti* | LS479444 |
| 35b | *F. equiseti* | KY466715 |
| D90 | *F. equiseti* | KX966241 |
| Bo038NY-07 | *Bipolaris oryzae* | JF521650.1 |

**Note:** EF1-α, the translation elongation factor 1-α.
